# Blood α-Tocopherol, γ-Tocopherol Levels and Risk of Prostate Cancer: A Meta-Analysis of Prospective Studies

**DOI:** 10.1371/journal.pone.0093044

**Published:** 2014-03-25

**Authors:** Ran Cui, Zhu-Qing Liu, Qing Xu

**Affiliations:** Department of Medical Oncology, Shanghai Tenth People's Hospital, Tongji University, School of Medicine, Shanghai, China; Innsbruck Medical University, Austria

## Abstract

**Background:**

Epidemiological studies that have examined the association of blood α-tocopherol and γ-tocopherol (the principal bioactive form of vitamin E) levels with the risk of prostate cancer have yielded inconsistent results. In addition, a quantitative assessment of published studies is not available.

**Methods and Findings:**

In this meta-analysis, relevant studies were sought by a search of the PubMed and Embase databases for articles published up to October 2013, with no restrictions. Bibliographies from retrieved articles also were scoured to find further eligible studies. Prospective studies that reported adjusted relative risk (RR) estimates with 95% confidence intervals (CIs) for the association between blood tocopherol levels and the risk of prostate cancer were included. Nine nested case–control studies involving approximately 370,000 participants from several countries were eligible. The pooled RRs of prostate cancer for the highest versus lowest category of blood α-tocopherol levels were 0.79 (95% CI: 0.68–0.91), and those for γ-tocopherol levels were 0.89 (95% CI: 0.71–1.12), respectively. Significant heterogeneity was present among the studies in terms of blood γ-tocopherol levels (*p* = 0.008) but not in terms of blood α-tocopherol levels (*p* = 0.33). The risk of prostate cancer decreased by 21% for every 25-mg/L increase in blood α-tocopherol levels (RR: 0.79; 95% CI: 0.69–0.91).

**Conclusions:**

Blood α-tocopherol levels, but not γ-tocopherol levels, were inversely associated with the risk of prostate cancer in this meta-analysis.

## Introduction

Vitamin E is an important chain-breaking antioxidant that prevents free radical reactions and lipid peroxidation_ENREF_1[1]. Owing to this antioxidant function, vitamin E may be particularly relevant in relation to the development of several cancers, including prostate cancer_ENREF_2[2]. α-Tocopherol is the predominant form of vitamin E in the plasma, regardless of dietary intake, due to preferential binding by the hepatic α-tocopherol transfer protein[3], [4]._ENREF_5 This molecule protects cell membranes and DNA from free radical damage that may lead to malignant transformation[1], [5]. The major food sources of α-tocopherol include vegetable oils such as sunflower seed oil, and that of γ-tocopherol are vegetable oils such as soy and maize oil_ENREF_2_ENREF_3_ENREF_3. γ-Tocopherol, which is the predominant vitamin E isoform consumed in the United States[6], has several anti-carcinogenic properties that are distinct from those of α-tocopherol. For instance, γ-tocopherol and its primary metabolite 2,7,8-trimethyl-2-(h-carboxyethyl)-6-hydroxychroman, exhibit anti-inflammatory activities via the inhibition of cyclooxygenase-2 activity_ENREF_4._ENREF_5 Many epidemiological studies have evaluated the relationship of blood α- and γ-tocopherol levels with the risk of prostate cancer; however, their results were equivocal, modest, or null.

The principal objective of this review was to evaluate the evidence from prospective studies on blood levels of α- and γ-tocopherols and the risk of prostate cancer, by summarizing it quantitatively with a meta-analysis approach.

## Methods

### Search strategy and eligibility criteria

This meta-analysis was conducted in accordance with PRISMA (Preferred Reporting Items for Systematic Reviews and Meta-Analyses) guidelines[7]_ENREF_6. We conducted a literature search of articles published before October 2013 in the PubMed and Embase databases. We used the following search terms without restrictions: “vitamin E”, “tocopherols”, “micronutrients”, and “prostate cancer”. Moreover, we reviewed the reference lists of retrieved articles to identify any studies that were not identified from the preliminary literature searches. Studies were included in the meta-analysis if they met the following criteria: (1) they had a prospective design (cohort or nested case–control studies); (2) the exposure of interest was blood (plasma or serum) levels of α- and γ-tocopherols; (3) the outcome of interest was prostate cancer; and (4) adjusted relative risk (RR) estimates with 95% confidence intervals (CIs) were reported. Where data sets overlapped or were duplicated, only the study with the largest number of cases was included.

### Quality assessment

Before data extraction and synthesis, we conducted a critical quality assessment on preliminarily included studies, by using the 9-star Newcastle–Ottawa Scale (NOS)[8]. This scale includes three aspects of evaluation: selection, comparability, and exposure between the case group and control group. Studies that scored <5 stars would be excluded. Two authors (R.C. and Z.Q.L.) independently assessed the quality of nine studies. Any discrepancies in NOS items between the two authors were resolved by consensus.

### Data extraction

Two authors independently extracted and cross-checked the data to reach a consensus. The following variables were recorded: the first author's last name, publication year, country where the study was performed, study period, participant age, sample size (cases and controls or cohort size), measure and range of exposure, variables adjusted for in the analysis, and RR estimates with corresponding 95% CIs for the highest versus lowest categories of blood α- and γ-tocopherol. Given the confounding effect of covariates on the meta-analysis, we extracted the RRs that reflected the greatest degree of control for potential confounders for use in the primary analysis.

### Data synthesis and statistical analysis

Study-specific RR estimates were combined using a random-effects model, which considers both within-study and between-study variation[9]. Statistical heterogeneity among studies was evaluated with the Q and I^2^ statistics[10]. Sensitivity analyses evaluated whether the results could have been markedly affected by a single study. Subgroup analyses were performed for geographic area, study quality, and range of exposure. Potential publication bias was evaluated using the Egger regression asymmetry test[11]. For the meta-analysis of the dose–response relationship between blood α-tocopherol levels and prostate cancer risk, we used the method of generalized least squares for trend estimation, as proposed by Greenland and Longnecker[12]_ENREF_11 and Orsini et al.[13]._ENREF_12_ENREF_12 Using this method, we computed the trend from the correlated natural logarithms of RR estimates across categories of α-tocopherol levels. For each study, the median level of α-tocopherol for each category was assigned to each corresponding RR estimate. We examined a potential nonlinear dose–response relationship between α-tocopherol levels and prostate cancer by modeling α-tocopherol levels using restricted cubic splines with three knots at percentiles 25%, 50%, and 75% of the distribution[14]. A *p* value for nonlinearity was calculated by testing the null hypothesis that the coefficient of the second spline is equal to 0. All statistical tests were performed using Stata 12 (Stata Corp., College Station, Texas). A *p* value of <0.05 was considered statistically significant.

## Results

### Literature search

**Figure 1:**
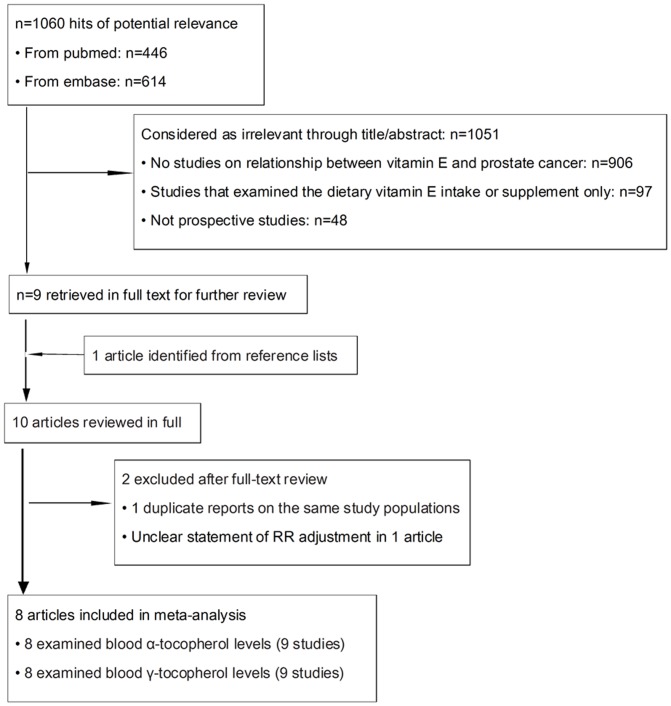
Literature search and study selection.

A flow diagram of our literature search is shown in Figure 1. In brief, we identified 10 potentially relevant articles concerning blood α- and γ-tocopherol levels in relation to the risk of prostate cancer. Two articles[15], [16]_ENREF_13_ENREF_14 were excluded because of unclear statement of RR adjustment and _ENREF_14reporting on the same population as in another study[17]_ENREF_14. The remaining eight articles, including nine studies (one article[17] reported results from two subcohorts) on blood α- and γ-tocopherol levels were included in the meta-analysis.

### Study characteristics

All nine studies were nested case–control studies and published between 1999 and 2012 (Table 1); they involved a total of 4,004 cases and 6,890 controls. Seven studies[17]–[22] _ENREF_17were conducted in the United States, and two in Europe[23], [24]._ENREF_19 Two studies[21], [24] measured plasma α- and γ-tocopherol levels, and seven studies[17]–[20], [22], [23] measured serum α- and γ-tocopherol levels. α-Tocopherol and γ-tocopherol levels were assayed using high-performance liquid chromatography.

**Table 1:** Characteristics of prospective studies on blood α- and γ-tocopherol levels and risk of prostate cancer.

Source	Location	Study period	study type	Age, y (mean±SD)	No. of Cases	No. of matched controls	No. of participants	Measure/Range of Exposure (mg/L)	Adjustment for Covariates
Weinstein et al, 2012	United States	1993–2001	Nested case-control study	55–74	680	824	28,243	Serum α-tocopherol: ≤12.3(Q1),>24.5(Q5); Serum γ-tocopherol: ≤1.38(Q1),>4.78(Q5)	Age, time since initial screening,, year of blood draw, study center, serum cholesterol, and serum β-carotene.
Gill et al, 2009	United States	1993–1996	Nested case-control study	45–75	467	936	96,382	Serum α-tocopherol: 9.0(Q1),25.1(Q4); Serum γ-tocopherol:0.6(Q1),3.4(Q4)	Age, fasting hours prior to blood draw, BMI, family history of prostate cancer, and education level.
Key et al, 2007	European countries	1992–2000	Nested case-control study	Cases:60.4(5.8) Controls:60.1(5.7)	966	1,064	137,001	Plasma α-tocopherol:<11.32(Q1), ≥16.80(Q5); Plasma γ-tocopherol:<0.63(Q1), ≥1.61(Q5)	BMI, smoking status, alcohol intake, physical activity level, marital status, and educational level.
Huang et al, 2002	United States	CLUE ^*^ I cohort (1974–1996); CLUE II cohort (1989–1996)	Nested case-control study	CLUE I:Cases:54(9) Controls:54(9) CLUE II: Cases:66 (8) Controls:66 (8)	CLUE I 182 CLUE II 142	CLUE I 364 CLUE II 284	CLUE I 9,804 CLUE II 10,456	CLUE I: Serum α-tocopherol: 9.6(Q1),15.5(Q5); Serumγ-tocopherol:1.6(Q1),3.5(Q5), CLUE II: Serum α-tocopherol:10.4(Q1),17.5(Q5); Serum γ-tocopherol:1.8(Q1),4.1(Q5)	Age, number of years since blood was drawn, disease stage at diagnosis, smoking status, and BMI.
Goodman et al, 2003	United States	1983–1997	Nested case-control study	45–74	205	483	18,314	Serum α-tocopherol:10.67(Q1),16.80(Q4); Serum γ-tocopherol:1.75(Q1),3.57(Q4)	Age, study center at randomization, smoking status, and year of randomization.
Gann et al, 1999	United States	1982–1995	Nested case-control study	40–84	578	1,294	22,071	Plasma α-tocopherol:8.56(Q1),14.44(Q5); Plasma γ-tocopherol: 1.25(Q1),2.53(Q5)	Physical activity, BMI, plasma total cholesterol, alcohol consumption, and multivitamin supplement use.
Weinstein et al, 2005	Finland	1985–1988	Nested case-control study	50–69	100	200	29,133	Serum α-tocopherol:12.6(T1),15.78(T3); Serum γ-tocopherol: 0.76(T1),1.08(T3)	Age, BMI, height, smoking status, benign prostatic hyperplasia, physical activity, urban residence, education, marital status, and serum cholesterol
Cheng et al, 2011	United States	1985–2005	Nested case-control study	Cases:60.6(5.7) Controls:60.3(5.8)	684	1,441	18,314	Serum α-tocopherol:11.16(Q1),32.84(Q4); Serum γ-tocopherol: 0.99(Q1),9.15(Q4)	Age, race, randomization assignment, family history of prostate cancer in first-degree relatives, alcohol consumption, smoking status, BMI, and serum cholesterol

Abbreviations: BMI: body mass index, T:tertile, Q:quartile/quintile, SD: standard deviation. * Derived from the slogan of a campaign, “Give us a CLUE to cancer.”

Most studies provided risk estimates that were adjusted for age (seven studies), smoking status (six studies), and body mass index (seven studies); few adjusted for alcohol consumption (three studies), blood cholesterol (three studies), and physical activity (three studies)._ENREF_20 All articles but one[23] _ENREF_18stated the follow-up duration in the text. The mean NOS score was 7.3 stars (range, 6–8 stars; Table 2). Therefore, the overall quality of all studies was fair.

**Table 2:** Methodological quality assessment based on the NOS.^a^

Source	Selection				Comparability^f^	Exposure			
	Definition^b^	Representativeness^c^	Selection^d^	Definition^e^		Ascertainment^g^	Method^h^	Rate^i^	Total^j^
Weinstein et al, 2012	1	1	1	1	1	1	1	0	7
Gill et al, 2009	1	1	1	1	2	1	1	0	8
Key et al, 2007	1	1	1	1	2	1	1	0	8
Huang et al, CLUE I 2002	1	1	1	1	2	1	1	0	8
Huang et al, CLUE II 2002	1	1	1	1	2	1	1	0	8
Goodman et al, 2003	1	1	1	1	1	1	1	0	7
Gann et al, 1999	1	1	1	0	1	1	1	0	6
Weinstein et al, 2005	1	1	1	0	1	1	1	0	6
Cheng et al, 2011	1	1	1	1	2	1	1	0	8

a Assessed with the 9-star Newcastle-Ottawa Scale(NOS).

b Adequate definition of cases(0,1star).

c Consecutive or obviously representative series of cases (0,1).

d Selection of controls: Community controls (0,1).

e Definition of controls: No history of disease (endpoint) (0,1).

f Study controls for the most important factor or any additional factor(0,1,2).

g Secure record (eg surgical records) (0,1).

h Same method of ascertainment for cases and controls(0,1).

i Same non-response rate for both groups(0,1).

j Total: minimum equals 1; maximum equals 9 stars.

### High vs. low α- and γ-tocopherol levels

**Figure 2:**
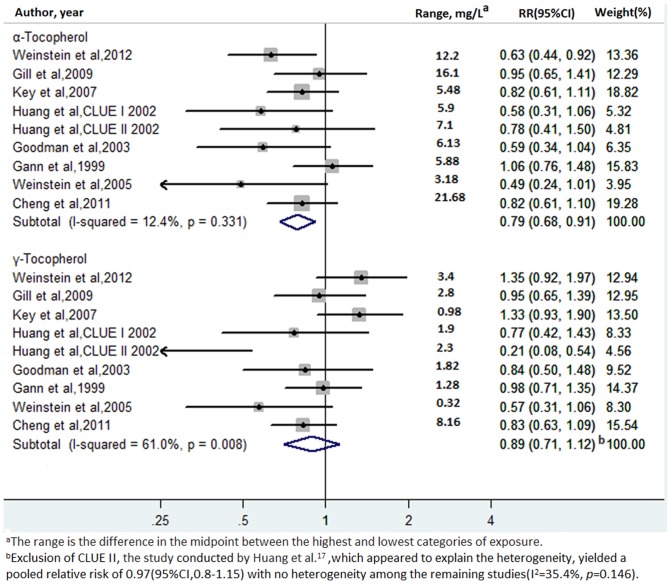
Adjusted relative risks of prostate cancer for the highest vs. lowest categories of blood α- and γ-tocopherol levels.

Multivariate-adjusted RRs for each study and the highest vs. lowest categories of blood α- and γ-tocopherol levels in all studies are shown in Figure 2. The pooled RRs of prostate cancer for the highest vs. lowest categories of α-tocopherol and γ-tocopherol levels were 0.79 (95% CI: 0.68–0.91) and 0.89 (95% CI: 0.71–1.12), respectively. There was statistically significant heterogeneity among the studies in the case of γ-tocopherol levels (I^2^: 61.0%, *p* = 0.008) but not in the case of α-tocopherol levels (I^2^: 12.4%, *p* = 0.33). The Egger test showed no evidence of publication bias for α-tocopherol levels (*p* = 0.08) or γ-tocopherol levels (*p* = 0.08).

### Sensitivity and stratifying analyses

A sensitivity analysis was conducted to explore the heterogeneity among the studies in the case of γ-tocopherol levels and prostate cancer risk. When we omitted the studies one by one and repeated the meta-analysis, the results were not substantially changed. However, exclusion of CLUE II, the study by Huang et al.[17],_ENREF_15 yielded a pooled RR of 0.97 (95% CI: 0.8–1.15), with no heterogeneity among the remaining studies (I^2^: 35.4%, *p* = 0.15). This study[17] was the only one that showed a statistically significant inverse association between γ-tocopherol levels and prostate cancer and appeared to explain the heterogeneity among the studies.

Upon stratification by geographic region, we found that the pooled RRs of blood α-tocopherol levels in relation to the risk of prostate cancer were 0.80 (95% CI: 0.67–0.95) for studies conducted in the United States and 0.71 (95% CI: 0.45–1.12) for studies conducted in Europe. There was no statistically significant heterogeneity among the studies in the case of α-tocopherol levels (United States, I^2^: 18.1% and *p = *0.29; Europe, I^2^: 40.5% and *p = *0.20). Stratification by the range of exposure showed no statistical difference between the narrow (≤12 mg/L difference in median intake between the highest and lowest categories) and wide (>12 mg/L difference) ranges of exposure; the RRs of blood α-tocopherol levels and prostate cancer for these ranges were 0.77 (95% CI: 0.62–0.97) and 0.79 (95% CI: 0.64–0.98), respectively. No statistical heterogeneity was observed among the studies in the case of α-tocopherol levels for the two ranges (≤12 mg/L difference, I^2^: 25.9% and *p = *0.24; >12 mg/L difference, I^2^: 16.0% and *p = *0.30). No statistical difference was found when we stratified the studies by methodological quality. The RR of α-tocopherol levels in relation to prostate cancer in studies that met higher quality criteria (8 stars) was 0.82 (95% CI: 0.69–0.97), and that of studies that met lower quality criteria (6–7 stars) was 0.71 (95% CI: 0.50–1.01).

### Dose–response meta-analysis

**Figure 3:**
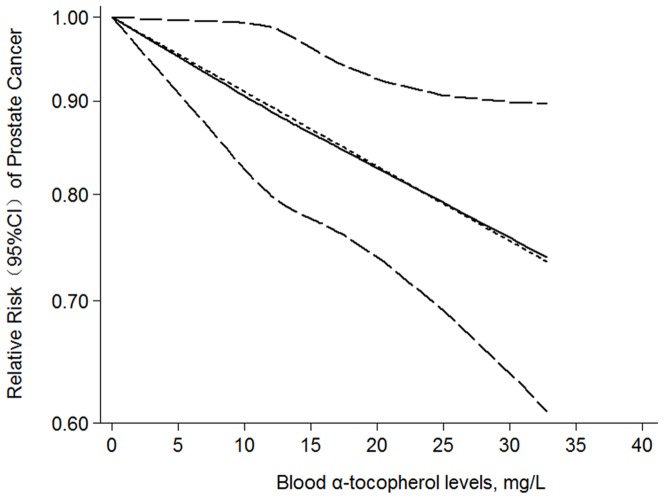
Dose–response relationship between blood α-tocopherol levels and relative risk of prostate cancer. Blood α-tocopherol levels were modeled with a linear trend in a random-effects meta-regression model. The solid line represents point estimates of association between blood α-tocopherol levels and prostate cancer risk; dashed lines are 95% confidence intervals (CIs).

We next assessed the dose–response relationship between blood α-tocopherol levels and the risk of prostate cancer, using all available data points from each study. The test for a nonlinear dose–response relationship yielded significant results (*p*<0.05). However, we detected a slight curvature in the relationship curve (Figure 3). Furthermore, we found that the between-study standard deviation was very close to zero (√tau^2^ = √0.0000078 = 0.003), suggesting that the study-specific trends had only a small spread around the average trend (coefficient  = −0.0095) for the nine studies. In addition, the goodness-of-fit test (*χ^2^* = 8.81, *p* = 0.36) implied no further problems with the fitted model. A 25-mg/L increment in blood α-tocopherol conferred an RR of 0.79 (95% CI: 0.68–0.91, *p* = 0.002).

## Discussion

Our results from the meta-analysis of nested case–control studies suggest that increased blood α-tocopherol levels, rather than γ-tocopherol levels, are inversely associated with the risk of prostate cancer. Overall, the risk of prostate cancer decreased by 21% for every 25-mg/L increase in blood α-tocopherol level.

In the Alpha-Tocopherol, Beta-Carotene Cancer Prevention (ATBC) study, Heinonen et al.[25] investigated the effect of α-tocopherol and β-carotene supplementation, separately or together, on the risk of prostate cancer in male smokers. They found that long-term supplementation with α-tocopherol substantially reduced prostate cancer incidence by 32% (95% CI: 12%–47%) and mortality by 41% (95% CI: 1%–65%). Similarly, observational data also suggested a vitamin E–prostate cancer–smoking interaction, with a beneficial association for vitamin E supplementation and high tocopherol levels in smokers for aggressive, but not non-aggressive, disease[22], [26]. In our study, we took the smoking status into consideration; we omitted three studies[18], [20],[21] that did not adjust the RR for smoking and repeated the meta-analysis of α-tocopherol in relation to prostate cancer risk. However, the results did not change appreciably (RR: 0.75, 95% CI: 0.63–0.85), and no heterogeneity was observed (I^2^: 0%, *p* = 0.63).

The nested case–control design potentially offered impressive reductions in costs and efforts of data collection and analysis compared with the full cohort approach, with relatively minor loss in statistical efficiency[27]. A strength of this study is that our quantitative assessment was based on biological samples collected at the baseline prior to disease development, which was considered more objective and would better minimize recall bias and miscalculation compared with cohort studies[26], [28] using self-reported food frequency questionnaires to obtain information about the doses of dietary vitamin E or supplements. Recall bias and miscalculation might exaggerate or underestimate the risk estimates in these cohort studies.

Our study also has several limitations. First, the current meta-analysis is unable to solve problems with confounding factors that could be inherent in the included studies. Although most studies adjusted for other possible risk factors for prostate cancer, residual or unknown confounding cannot be excluded as a potential explanation for the observed findings. A second limitation is that our results are likely to be affected by the wide range of values for the cutoff points for the lowest and highest categories for α- and γ-tocopherol levels in several studies, which might also impact the current analysis. Third, heterogeneity may be introduced because of methodological differences among studies, including different ranges of exposure and inadequate or unreported follow-up duration or failure to follow up cases. Finally, potential publication bias could be of concern because small studies with null results tended not to be published, especially in the case of clinical trials. In our meta-analysis, we found no evidence of publication bias. However, we will update our study when possible.

Although blood α-tocopherol levels were associated with reduced prostate cancer risk in one prospective study[18] and although this association has also been proved in this meta-analysis, the results from one population-based prospective study raised the possibility that only serum γ-tocopherol was associated with prostate cancer risk[17]. This is because the high dose of vitamin E supplement intake or high plasma α-tocopherol levels could reduce plasma γ-tocopherol concentration[18], [29]. In other words, the potential role of γ-tocopherol in reducing the risk of prostate cancer might be masked or weakened by α-tocopherol. Despite the higher dietary intake of γ-tocopherol, circulating concentrations of α-tocopherol are far higher than those of γ-tocopherol[30]. In fact, in our meta-analysis, among the nine studies on the association of blood γ-tocopherol concentrations with prostate cancer risk, seven showed an inverse association, which was statistically significant in one study[17], though no beneficial effect was found. γ-Tocopherol may contribute significantly to human health in ways that have not yet been recognized, it is our opinion that this possibility should be considered and carefully evaluated. With present evidence, we suggest that people, especially the aged, should intake sufficient vegetables and fruits that are enriched in Vitamin E such as lettuce and kiwi fruit in daily life. We do not recommend the healthy population to intake excess vitamin E pills or its elemental diets. Given the interactions between α- and γ-tocopherols, further study is warranted.

## Supporting Information

Checklist S1 (PDF)
